# The Roles of Alix and VPS4A in Autophagy and Endosomal Pathways and Their Relation to HBV Replication

**DOI:** 10.1096/fj.202504742R

**Published:** 2026-04-08

**Authors:** Jia Li, Thekla Kemper, Shuping Tong, Xueyue Wang, Yong Lin, Mengji Lu

**Affiliations:** ^1^ Institute for Virology University Hospital Essen, University of Duisburg‐Essen Essen Germany; ^2^ Department of Laboratory Medicine, West China Hospital Sichuan University Chengdu China; ^3^ Liver Research Center, Rhode Island Hospital, the Warren Alpert School of Medicine Brown University Providence Rhode Island USA; ^4^ State Key Laboratory for Diagnostic and Treatment of Infectious Diseases, the First Affiliated Hospital, School of Medicine Zhejiang University Hangzhou China; ^5^ The Key Laboratory of Molecular Biology of Infectious Diseases Designated by the Chinese Ministry of Education Chongqing Medical University Chongqing China

**Keywords:** Alix, autophagosome, degradation, endosomal trafficking, HBV, VPS4A

## Abstract

Autophagic and endosomal pathways coordinately contribute to hepatitis B virus (HBV) production, with the endosomal sorting complex required for transport (ESCRT) components ALG‐2‐interacting protein X (Alix) and the vacuolar protein sorting 4A (VPS4A) playing important but mechanistically elusive roles. This study investigates the roles of Alix and VPS4A in HBV biogenesis within the context of endosomal trafficking and autophagy. Using gene silencing and overexpression of wild‐type (WT) or dominant‐negative (DN) mutants of Alix and VPS4A in HBV replication cell models, we found that Alix silencing increased intracellular HBV DNA and HBV surface antigen (HBsAg), extracellular HBsAg, and virions, while decreasing secreted naked capsids. It promoted HBsAg secretion along the early endosomes but reduced its transport to late endosomes and autophagosomes. Furthermore, Alix silencing impaired autophagosome formation by activating the AKT/MTOR pathway. In contrast, VPS4A silencing had minimal effects, whereas DN VPS4A significantly blocked HBV secretion by disrupting endosomal trafficking, promoting autophagosome formation and lysosome activity, ultimately leading to HBV degradation. Our findings demonstrate that the endosomal pathway is critical for HBV secretion when lysosomal activity is suppressed. Conversely, increased lysosomal function drives HBV degradation through the autophagosome‐lysosome pathway.

## Graphical abstract

Alix and VPS4A function in HBV biogenesis and degradation in the context of the endosomal and autophagic pathways. Alix and VPS4A orchestrate HBV secretion and degradation via endosomal and autophagic pathways. Under normal conditions, ESCRT ensures efficient virion budding and MVB formation. Alix silencing impairs cargo sorting into MVBs and autophagosomes, leading to intracellular HBV accumulation. Conversely, dominant‐negative VPS4A triggers a global ESCRT collapse, blocking viral egress and redirecting components into autophagosomes and late endosomes for lysosomal degradation. Thus, the ESCRT complex acts as a critical switch balancing HBV secretion and eradication.

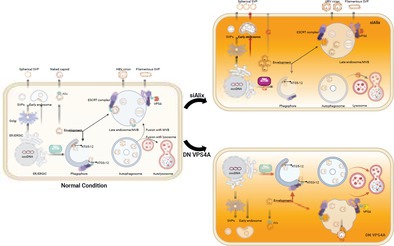

**Abbreviations**
Alix: ALG‐2‐interacting protein X
ATG: Autophagy‐related
CQ: Chloroquine
DN: Dominant‐negative
ESCRT: Endosomal sorting complexes required for transport
ER: Endoplasmic reticulum
EGF receptor (EGFR): Epidermal growth factor receptor
FBS: Fetal Bovine Serum
HBV: Hepatitis B Virus
HBcAg: HBV core protein
HBsAg: HBV surface antigen
IF: Immunofluorescence
LAMP1: Lysosomal‐associated membrane protein 1
MAP1LC3B/LC3B: microtubule associated protein 1 light chain 3 beta
MTOR: Mechanistic target of rapamycin kinase
MVB: Multivesicular body
PDI: protein disulfide isomerase
AKT: Protein kinase B
RPS6KB1: Ribosomal Protein S6 Kinase B1
SQSTM1/p62: Sequestosome 1
siRNA: Small interfering RNA
SVP: Subviral particle
VPS4: Vacuolar protein sorting 4
WT: Wild‐type

## 1 Introduction

Hepatitis B Virus (HBV) remains a major global health concern, with approximately 296 million chronically infected people worldwide [1]. Chronic HBV infection can lead to severe liver diseases, including cirrhosis and hepatocellular carcinoma, collectively resulting in over 800 000 deaths annually. The replication of HBV relies on its intricate co‐option of host cellular machinery, and the endosomal sorting complexes required for transport (ESCRT) system is essential for its assembly and virion production [2].

The ESCRT machinery is an ancient system for membrane remodeling and scission. It consists of the peripheral membrane protein complexes ESCRT‐0, ‐I, ‐II, ‐III, vacuolar protein sorting 4 (VPS4) ATPase, and the ALG‐2‐interacting protein X (Alix) homodimer [3]. Enveloped viruses, including Ebola, Human Immunodeficiency Virus, and HBV, utilize ESCRT for their budding [2, 4, 5]. Within this pathway, Alix and VPS4 play distinct yet pivotal roles. Alix acts early, serving as an adaptor and nucleating factor, while VPS4 acts late, using ATP hydrolysis to disassemble and recycle the ESCRT‐III complex [6, 7]. Human Alix consists of three regions: the N‐terminal Bro1 domain, the central V domain, and the C‐terminal proline‐rich region [8]. Alix and its Bro1 domain facilitate the egress of HBV naked capsids [9, 10], yet the precise mechanisms remain unclear. The regulatory functions of VPS4A in the HBV lifecycle remain controversial. Initial studies by Kian Chua et al. [11] demonstrated that VPS4A dominant‐negative mutants (DN VPS4A) potently suppress HBV replication. However, in other experimental settings, the DN VPS4A arrested HBV virion release by impairing multivesicular body (MVB) biogenesis but did not affect spherical HBV surface antigen (HBsAg) secretion [12, 13], suggesting a complex and selective mechanism of action [14]. How these distinct roles of Alix and VPS4A converge to regulate critical processes such as HBV replication, assembly, and release is therefore a key unresolved question.

Autophagy represents another critical pathway involved in the HBV lifecycle [15]. Intriguingly, the ESCRT machinery is also integral to autophagosome formation and maturation, with components like VPS4 and Alix being required for efficient autophagic flux. VPS4 silencing prevents autophagosome closure and fusion with lysosomes [16, 17, 18]. Alix silencing impairs basal autophagic flux through interactions with the autophagy‐related (ATG) 12‐ATG3 complex [19]. This convergence of the MVB and autophagy pathways, both reliant on ESCRT function, presents a compelling, unified context for understanding HBV egress. This prompted our investigation into how Alix and VPS4A regulate the autophagy and endosomal pathways during HBV replication and its subsequent impact on HBV production and distribution.

In the present study, we explored the distinct roles of Alix and VPS4A in the HBV life cycle. Specifically, the question was addressed how Alix and VPS4A influence endosomal, autophagic, and lysosomal functions and change trafficking of HBV components along different cellular compartments, thereby affecting viral assembly and release. Using gene silencing and overexpression in cell model systems, we demonstrate that these factors act as sequential gatekeepers in a host pathway that determines the fate of HBV. Our results thus shed light on a critical regulatory mechanism within the ESCRT pathway that balances viral secretion with lysosomal degradation.

## 2 Materials and Methods

### 2.1 Cell Culture

All used cell lines were cultured at 37°C in a humidified environment containing 5% CO2. HepG2.2.15 human hepatoma cells, harboring integrated dimers of the HBV genome (GenBank accession number, U95551), were cultured in RPMI 1640 medium (Gibco, 11 875 093), supplemented with 10% inactivated fetal bovine serum (FBS, Gibco, 10 270 106), 100 U/mL of penicillin (Gibco, 15 140 122), 100 μg/mL of streptomycin (Gibco, 15 140 122), 1% non‐essential amino acids (NEAAs, Gibco, 111 500 500), 1% HEPES (Gibco, 15 630 080), and 500 μg/mL of G418 (ITW Reagents, A6798). The HBV transient transfection cell line Huh7 was grown in DMEM medium (Gibco, 41 966 029), supplemented with 10% inactivated FBS, 100 U/mL of penicillin, 100 μg/mL of streptomycin, 1% NEAAs, and 1% HEPES.

### 2.2 siRNAs and Plasmids

siNC (negative control, Allstars Negative Control siRNA, 1 027 280). siAlix (SI02655345) and siVPS4A (SI04302158), siRAB7 (SI02662240) were purchased from Qiagen. An HBV replication‐competent clone pSM2 harboring a head‐to‐tail tandem dimer of the HBV genome (GenBank accession number: V01460) was provided by Dr. Hans Will (Heinrich‐Pette‐Institute, Hamburg, Germany) [20]. The plasmid information is shown in Table S1. The primers of ACTB (QT01680476), Alix (QT00067942), and VPS4A (QT00022029) were purchased from Qiagen. Transfection of plasmid or small interfering RNAs (siRNAs) was performed by using Lipofectamine 2000 transfection reagent (Invitrogen, 11 668 019) according to the manufacturer's instructions.

### 2.3 Chemical Reagents

AKTi (124017), Rapamycin (R8781), and chloroquine (CQ, C6628) were purchased from Sigma‐Aldrich. EBSS (14155048) was purchased from Thermo Fisher. pHrodo Red Epidermal Growth Factor (EGF) Conjugate (P35374) was purchased from Invitrogen. Western blot assay was carried out as described previously [21]. The signals were visualized with an enhanced chemiluminescence (ECL) system (GE Healthcare, 12 316 992). The relative levels of indicated proteins were determined by quantifying the gray scales of bands using ImageJ software: First, we measured the grayscale value for each specific protein by using the software ImageJ and calculated the ratio to its corresponding ACTB grayscale value, yielding the relative expression level of each treated protein normalized to ACTB protein. Next, we used the calculated relative expression level for each protein and compared the levels between the treated and untreated samples. Antibodies used in the experiments are listed in Table S2.

### 2.4 Detection of HBV Gene Expression and Replication

HBsAg and HBeAg levels in the supernatants and lysates were measured using a chemiluminescent microparticle immunoassay (CMIA, Abbott Diagnostics, 06C3622 and 06C3237).

Intracellular HBV DNA encapsidated in core particles was extracted as previously described [22]. Briefly, cells were lysed using a buffer containing 50 mM Tris–HCl, 1 mM EDTA, and 1% NP‐40 (Sigma–Aldrich, 74 385) at 4°C for 10 min. Cell debris and nuclei were removed by centrifugation, and the cell lysates containing HBV capsids were collected. The lysates were then incubated with 10 mM MgCl_2_, 100 μg/mL DNase I (Roche, 10 104 159 001), and 10 μg/mL RNases (Qiagen, 1 072 588) at 37°C for 30 min to degrade the extraneous nucleic acids. The reaction was terminated by the addition of 25 mM EDTA. Subsequently, 0.5 mg/mL proteinase K (Qiagen, 19 133) and 1% sodium dodecyl sulfate (SDS) were added, and the mixture was incubated at 55°C for 2 h. The DNA was extracted using phenol/chloroform (1:1), precipitated with isopropanol, and washed with 75% ethanol.

HBV progeny DNA was extracted from cell culture supernatants using QIAamp DNA Blood Maxi Kit (Qiagen, 51 194) according to the manufacturer's instructions.

HBV Virions and HBV capsids from supernatants were immunoprecipitated with anti‐HBsAg (ZSGB‐BIO, ZM‐0122) or anti‐HBcAg (ZSGB‐BIO, ZA‐0121), respectively. The incubation was performed overnight at 4°C under rotation, followed by a 4‐h incubation with protein A/G Sepharose (Abcam, ab193262) at 4°C. The beads were sedimented by short centrifugation, washed three times with PBS, and the bound particles were then disrupted with buffer AL. After digestion of the precipitate with DNase I, HBV DNA was extracted from the immunoprecipitate and quantified using the QIAamp DNA Blood Kit and PCR.

Real‐time PCR was used to measure HBV DNA levels (Invitrogen, 11 733 046), using the primer pair 5′‐GTTGCCCGTTTGTCCTCTAATTC‐3′ (forward) and 5′‐GGAGGGATACATAGAGGTTCCTT‐3′ (reverse).

HBV RNA was extracted from cells using TRIzol reagent (Invitrogen, 15 596 018) followed by DNA digestion. Quantification of HBV RNA was performed via real‐time reverse transcription (RT)‐PCR (Qiagen, 204 154) using the primer pair 5′‐CCGTCTGTGCCTTCTCATCT‐3′ (forward) and 5′‐TAATCTCCTCCCCCAACTCC‐3′ (reverse). The mRNA levels were normalized to the ACTB (Qiagen, QT01680476) mRNA level.

### 2.5 Immunofluorescence (IF) Microscopy

Cells were grown on coverslips and treated as indicated in each experiment. After treatment, cells were fixed with 4% paraformaldehyde and permeabilized with 0.1% Triton X‐100 (Sigma, T8787). After washing and blocking, cells were incubated with the indicated primary antibodies at room temperature for 1 h. Afterward, the coverslips were washed and incubated with the corresponding secondary antibodies at room temperature for 1 h. Nuclei were stained with 6‐diamidino‐2‐phenylindole (DAPI, Carl Roth, 6843.1). The coverslips were then washed, mounted in an immunofluorescence mounting medium, and analyzed using an LSM710 microscope (Zeiss, Germany). The fluorescence intensity of the target proteins was quantified using ImageJ software. Colocalization efficiency, defined as the fraction of green fluorescent protein (GFP) pixels also positive for red pixels, was determined from randomly selected cells using ImageJ. Data were collected from at least five cells for each experiment. The primary antibodies used in IF staining are listed in Table S2.

### 2.6 Dye Quenched‐Bovine Serum Albumin (DQ‐BSA) Red Degradation Assay

The DQ‐BSA Red degradation assay was performed as previously described [23]. 48 h post‐treatment, cells were incubated with DQ‐BSA Red (10 μg/mL; Invitrogen, D12051) for 30 min. The fluorescent signal produced by lysosomal proteolysis of DQ‐BSA Red was quantified using an LSM 710 confocal microscope. In each experiment, fluorescence intensity was measured in at least 5 individual cells treated with DQ‐BSA. The average fluorescence intensity for the negative control was calculated and normalized to 1. The fold change in DQ‐BSA fluorescence intensity for each experimental sample was then determined relative to the negative control.

### 2.7 Detection of Lysosomal Activity

The lysosomal activity was detected by acridine orange (AO) staining or Lysotracker Red staining as described previously [23]. Briefly, treated cells were stained with 5 μM AO (Sigma, A9231) for 15 min or 50 nM Lysotracker Red (Thermo Fisher, L12492) for 30 min. After staining, cells were washed with PBS, fixed, and stained with DAPI. Fluorescent signals were captured at 488 nm (green) for AO or 594 nm (red) for Lysotracker Red using an LSM 710 confocal microscope.

### 2.8 Detection of Internalized EGF in Cells

The internalized EGF in cells was detected by the pHrodo Red Epidermal Growth Factor (EGF) conjugates (Invitrogen, P35374). Briefly, treated cells were stained with 50 ng/mL pHrodo Red EGF for 1 h. After staining, cells were washed with PBS, fixed, and stained with DAPI. Fluorescent signals were captured at 594 nm using an LSM 710 confocal microscope.

### 2.9 Statistical Analysis

Results are shown as the means ± SEM. Statistical analyses were performed using GraphPad Prism software (version 8; GraphPad Software Inc., La Jolla, CA). The difference of means between the two groups was determined by the Student's paired *t*‐test. Data for single‐factor experiments were performed by the one‐way ANOVA procedure. *p* < 0.05 was considered statistically significant and indicated by asterisks or pounds. All experiments were repeated at least three times independently.

Other detailed materials and methods are provided in the Supporting Information and Methods in the Supporting Information.

## 3 Results

### 3.1 Alix and VPS4A Affect HBV Replication

**FIGURE 1:**
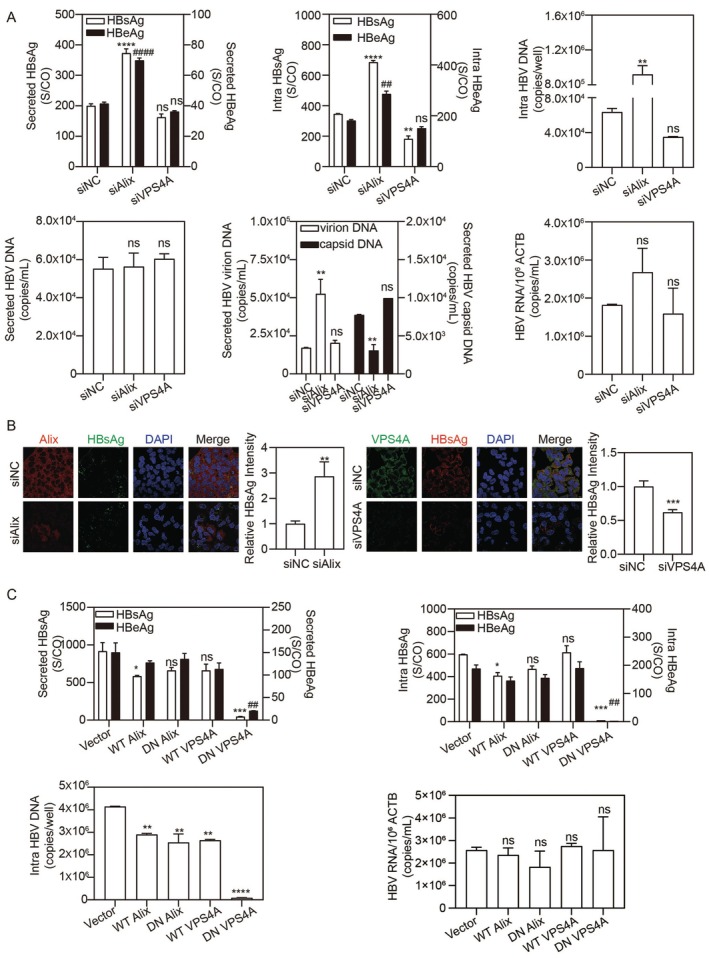
Alix and VPS4A functions are required for HBV production. HepG2.2.15 cells were transfected with siAlix, siVPS4A, or siNC for 72 h. (A) Secreted/intracellular HBsAg, HBeAg, HBV DNA, virions, capsids, and RNA levels were detected. (B) Intracellular distribution of HBsAg and Alix (or VPS4A) was visualized by immunofluorescence (IF) microscopy. The IF intensity of HBsAg was quantified using ImageJ software. Scale bar: 10 μm. (C) Huh7 cells were co‐transfected with HBV plasmid (pSM2) and WT or DN Alix/VPS4A plasmids for 48 h. Secreted/intracellular HBsAg and HBeAg, and intracellular HBV DNA/RNA were measured. *p < 0.05; **^,^^##^ *p* < 0.01; ***p < 0.001; ****^,^^####^ *p* < 0.0001; ns, not significant.

To investigate the crosstalk between HBV and the host ESCRT machinery, we examined whether viral expression alters the levels of Alix and VPS4A. Our results revealed that HBV plasmid transfection leads to a measurable up‐regulation of both Alix and VPS4A protein levels (Figure S1). This induction suggests that HBV actively modulates the ESCRT‐related pathways to optimize the host cellular environment for its life cycle. These findings are consistent with our previous observation that HBV upregulates RAB5A to facilitate the dual activation of endosomal and autophagic vesicle pathways for its replication [24]. To examine the role of Alix and VPS4A in HBV replication, HepG2.2.15, a harboring integrated dimer of the HBV genome cell line, was transfected with small interfering RNAs (siRNAs) targeting Alix (siAlix) or VPS4A (siVPS4A) or a negative control (siNC) for 72 h. The marked reduction of Alix and VPS4A expression by silencing was confirmed by real‐time PCR and western blot (Figure S2). Following Alix silencing, the levels of secreted and intracellular HBsAg and HBV e antigen (HBeAg), as well as intracellular HBV DNA increased (Figure 1A). Total secreted HBV DNA levels were not changed. Alix silencing significantly increased secreted HBsAg‐associated HBV DNA but decreased HBV core protein (HBcAg)‐associated HBV DNA, which mainly represents enveloped virions and capsids, respectively (Figure 1A). In contrast, VPS4A silencing had minimal impact on HBV replication, with only a slight decrease in accumulated HBsAg. Notably, neither Alix nor VPS4A silencing significantly affected HBV RNA levels, indicating that Alix and VPS4A primarily do not regulate HBV transcription (Figure 1A). Immunofluorescence (IF) staining further revealed that intracellular HBsAg expression levels increased with Alix silencing but decreased with VPS4A silencing (Figure 1B). These findings were consistent in Huh7 cells transfected with an HBV replication‐competent plasmid pSM2 (Figure S3). Thus, Alix and VPS4A intricately regulate HBV replication in hepatoma cells.

To further validate and dissect the roles of Alix and VPS4A in HBV production, we overexpressed wild‐type (WT) and dominant‐negative (DN) mutants of Alix (I212D) and VPS4A (E228Q) in pSM2‐transfected Huh7 cells. The I212D mutation in Alix is located in its Bro1 domain, which disrupts ESCRT protein recruitment and downstream membrane remodeling events and HBV assembly and secretion [9]. The E228Q mutation in VPS4A causes ATP hydrolysis deficiency, which impairs the disassembly and recycling of ESCRT complexes [11]. Overexpression of WT Alix significantly decreased both extracellular and intracellular HBsAg, whereas the DN Alix had no effect (Figure 1C). Like VPS4A silencing, WT VPS4A did not affect extracellular or intracellular HBsAg levels. However, DN VPS4A almost completely blocked HBV production in pSM2‐transfected Huh7 cells. All the plasmids markedly reduced intracellular HBV DNA levels without significant changes in HBV RNA levels (Figure 1C). Overall, Alix and VPS4A regulate HBV production in different ways. Subsequently, we examined the consequences of modulating these proteins in detail.

### 3.2 Alix and VPS4A Silencing Affect HBV Trafficking Along Endosomes and Autophagosomes

To understand the roles of Alix and VPS4A in HBV subcellular trafficking, we examined the colocalization of HBsAg with organelle markers: Protein Disulfide Isomerase (PDI) for ER, GOLGA2/GM130 for Golgi, RAB5A for early endosome, CD63 for late endosome, Microtubule Associated Protein 1 Light Chain 3 (LC3) for autophagosomes, and lysosomal‐associated membrane protein 1 (LAMP1) for lysosomes, following Alix and VPS4A silencing. Colocalization was quantified as the percentage of GFP‐positive pixels overlapping with the red organelle marker.

**FIGURE 2:**
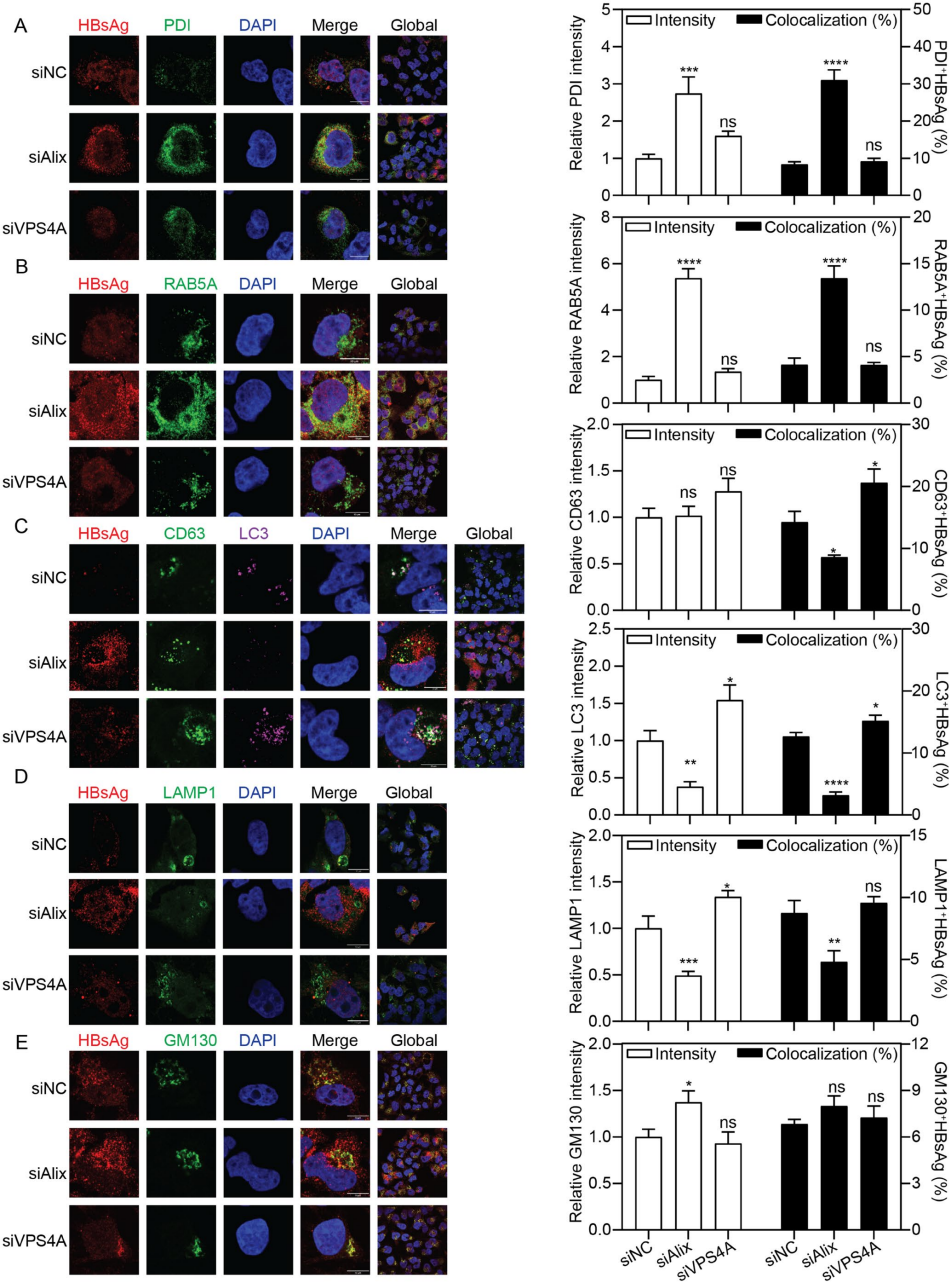
Alix and VPS4A silencing change the intracellular distribution of HBsAg. (A–E) HepG2.2.15 cells were transfected with siAlix, siVPS4A, or siNC, and harvested after 48 h. Subcellular distributions of HBsAg and (A) PDI, (B) RAB5A, (C) CD63, LC3, (D) LAMP1, and (E) GM130 were detected by IF with a confocal microscope. Scale bar: 10 μm. The IF intensity was analyzed using ImageJ software. The colocalization was determined in ImageJ by the green fluorescent protein pixels also positive for red pixels. *p < 0.05; **p < 0.01; ***p < 0.001; ****p < 0.0001; ns, not significant.

Alix silencing trapped HBsAg in the early secretory and endocytic compartments, as indicated by its increased retention within an expanded ER and early endosome (Figure 2A,B). In contrast, the progression of HBsAg beyond these compartments was significantly impaired. We observed a marked reduction in HBsAg colocalization with late endosomes, autophagosomes, and lysosomes, demonstrating a blockade in downstream trafficking to degradative organelles (Figure 2C,D). The Golgi apparatus, while expanded, showed no change in HBsAg association, suggesting it is not a major site of Alix‐dependent regulation (Figure 2E).

Unlike the pronounced changes observed with Alix silencing, VPS4A silencing left the early HBsAg pathway intact, causing no significant alteration in its colocalization with the ER (Figure 2A), early endosomes (Figure 2B), or Golgi (Figure 2E). The impact of VPS4A silencing became apparent at later compartments. It induced accumulation of late endosomes and autophagosomes, which was accompanied by a significant increase in HBsAg within these structures (Figure 2C). Although the lysosome was also expanded, the efficiency of HBsAg delivery to lysosomes was not enhanced (Figure 2D). Mechanistically, as VPS4A catalyzes the final scission of ESCRT‐III polymers, its loss likely causes a traffic jam at the late endosomal and autophagosomal stages. Together, these findings demonstrate that VPS4A silencing might not reroute HBsAg but instead delays its secretion by causing accumulation in late endosomes and autophagosomes.

We further validated the roles of Alix and VPS4A in Huh7 cells under transient HBV replication conditions. The overall trafficking phenotypes induced by gene silencing were consistent with those observed in HepG2.2.15 cells, despite cell line‐dependent variations in baseline colocalization levels and the magnitude of specific colocalization changes (Figure S4). Specifically, Alix silencing again enhanced HBsAg retention in the ER and impaired its delivery to degradative compartments. VPS4A silencing continued to show minimal impact on early trafficking but consistently delayed HBsAg secretion and promoted its association with late endosomes. These results confirm that the fundamental roles of Alix and VPS4A in HBV trafficking are conserved across different cellular models.

### 3.3 Alix and VPS4A Overexpression Divert HBV Trafficking to Distinct Endosomal Pathways

**FIGURE 3:**
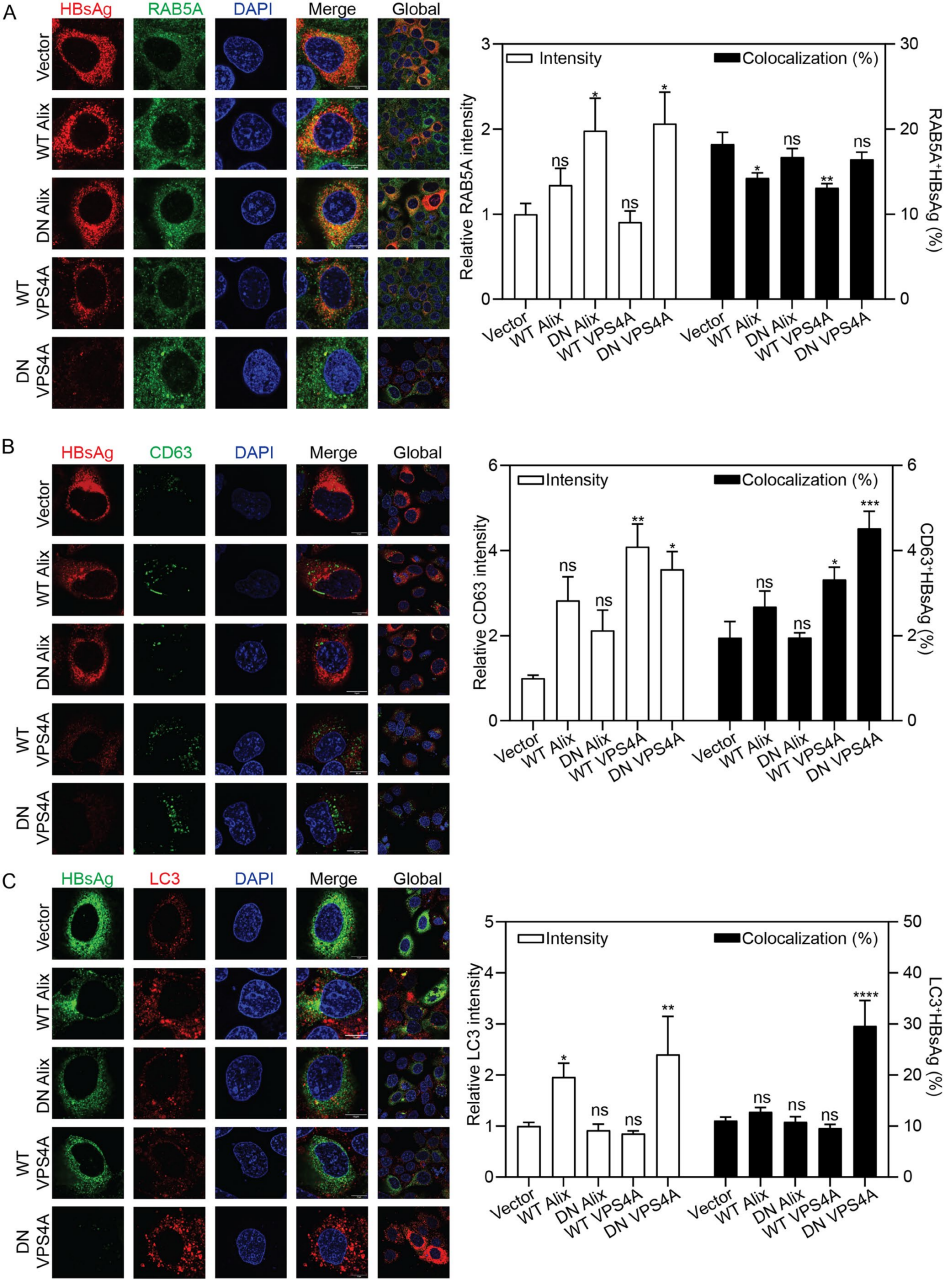
The expression of WT and DN mutants of Alix and VPS4A changes the intracellular distribution of HBsAg. Huh7 cells were transiently co‐transfected with pSM2 and WT or DN Alix/VPS4A plasmids. Subcellular distribution of HBsAg and (A) RAB5A, (B) CD63, and (C) LC3 was detected by IF with a confocal microscope. Scale bar: 10 μm. The IF intensity was analyzed using ImageJ software. The colocalization was determined in ImageJ by the green fluorescent protein pixels also positive for red pixels. *p < 0.05; **p < 0.01; ***p < 0.001; ****p < 0.0001; ns, not significant.

Next, we explored the consequences of Alix and VPS4A overexpression on HBV trafficking. We co‐transfected Huh7 cells with the pSM2 and expression plasmids encoding WT or DN Alix and VPS4A. WT Alix overexpression did not alter RAB5A IF intensity, but DN Alix induced RAB5A aggregates and increased its IF intensity (Figure 3A). Unlike Alix silencing, WT Alix overexpression significantly decreased its colocalization with HBsAg to 14%, while the I212D mutant exhibited no such effect (Figure 3A). These data suggest that functional Alix, but not the I212D residue, may regulate HBsAg trafficking to early endosomes, possibly through its specific protein domain [25]. Neither WT nor DN Alix altered late endosome formation or its association with HBsAg (Figure 3B). Although WT Alix increased LC3 levels, it did not promote HBsAg entry into autophagosomes (Figure 3C). These data demonstrate that Alix specifically regulates HBsAg at the early endosomal stage, while its role in late endosomal and autophagosomal trafficking appears to be minimal.

WT VPS4A did not alter early endosome formation (Figure 3A) but significantly increased late endosome formation (Figure 3B). Functionally, compared to the control, it reduced HBsAg colocalization with early endosomes to 13% and increased colocalization with late endosomes to 4%, respectively. DN VPS4A dramatically enhanced HBsAg colocalization with both late endosomes and autophagosomes (Figure 3C), which may facilitate its transport to the lysosome for subsequent degradation and ultimately leads to a sharp reduction in intracellular HBsAg levels.

In summary, Alix drives HBsAg trafficking through early endosomes, whereas VPS4A drives its progression to late compartments, with DN mutants diverting HBsAg toward degradative pathways.

### 3.4 Alix Silencing Impairs Autophagosome Formation

**FIGURE 4:**
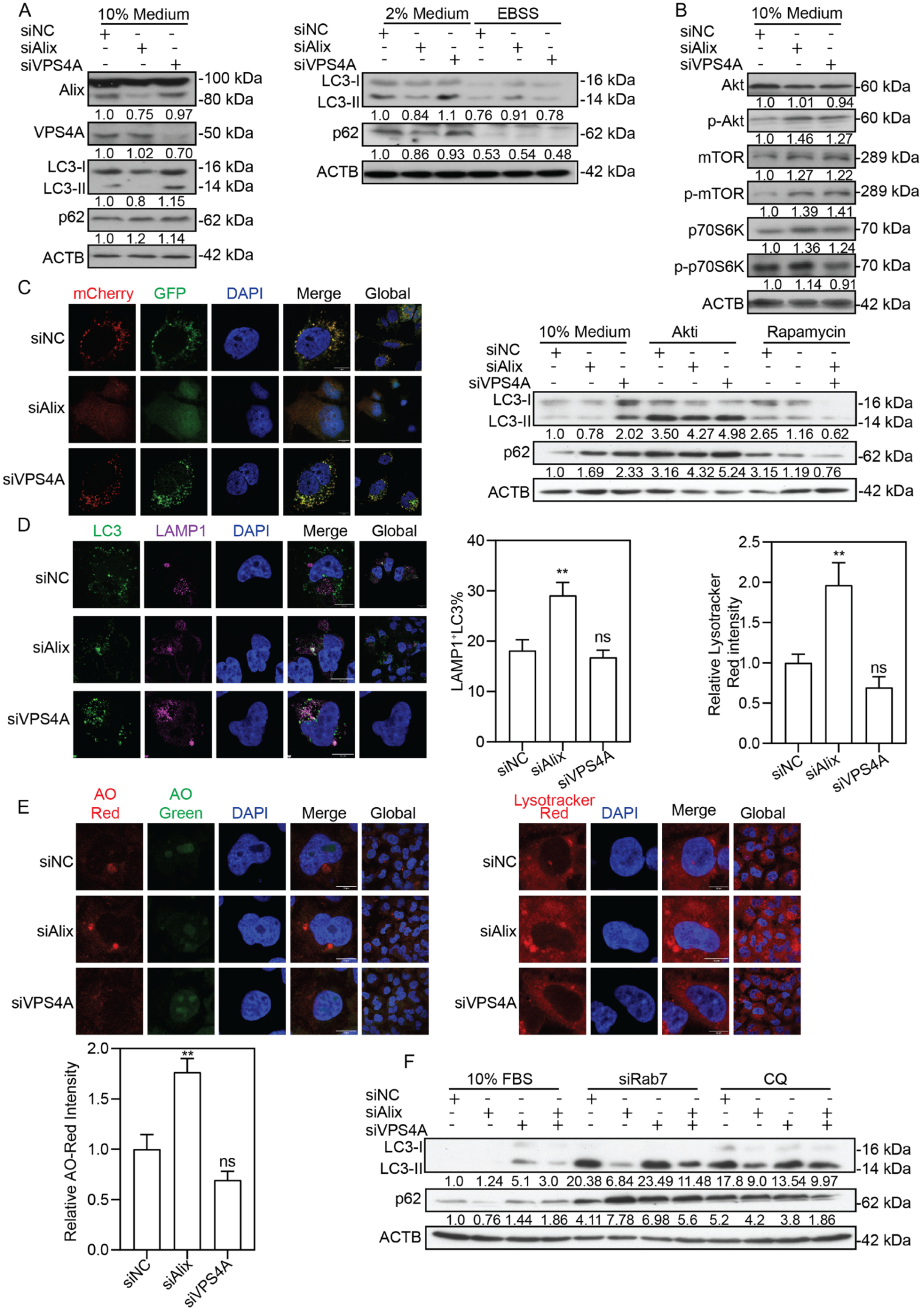
Alix silencing impairs autophagosome formation. (A) LC3 and p62 levels in HepG2.2.15 cells transfected with siAlix, siVPS4A, or siNC under serum modulation (10% vs. 2% FBS) or starvation (EBSS). (B) phosphorylated and total levels of Akt/mTOR/p70S6K in transfected cells, and LC3/p62 after Akt inhibitor (Akti) or rapamycin treatment. (C) Autophagic flux (mCherry‐GFP‐LC3 puncta) in Huh7 cells. Scale bar: 10 μm. (D) LC3‐LAMP1 colocalization in transfected cells. (E) Lysosomal acidity (acridine orange, AO; LysoTracker Red). Scale bar: 10 μm. (F) LC3/p62 levels after transfection with siRNAs alone, with siRab7, or CQ. **p < 0.01; ns, not significant.

Consistent with reports that Alix is necessary for basal but not starvation‐induced autophagy [19], our data revealed a nutrient context‐dependent role. Under 10% fetal bovine serum (FBS) (nutrient‐rich) and 2% FBS (reduced nutrients) conditions, Alix silencing decreased LC3 expression levels without a corresponding increase in sequestosome 1 (SQSTM1/p62), indicating suppressed autophagosome formation. In contrast, under Earle's balanced salt solution (EBSS) (starvation), Alix silencing slightly elevated LC3 levels (Figure 4A). These findings suggest that Alix primarily regulates autophagy under nutrient‐replete conditions.

To delineate the upstream mechanism, we examine the protein kinase B (AKT)/mammalian target of rapamycin (MTOR) pathway, a central regulator of autophagy [26]. Alix silencing robustly activated the AKT/MTOR pathway, as evidenced by increased phosphorylation of AKT (Ser473), MTOR, and its downstream target RPS6KB1/p70S6K (Figure 4B). To confirm the functional relationship between the AKT/MTOR pathway and the observed autophagic suppression, HepG2.2.15 cells treated with siRNAs were cultured with AKT inhibitor AKTi and MTOR inhibitor rapamycin for 72 h. Both inhibitors effectively restored LC3 expression levels in Alix‐silenced cells. Thus, Alix silencing impairs autophagy through the AKT/MTOR signaling pathway.

Intriguingly, VPS4A silencing induced LC3 accumulation in the presence of FBS (Figure 4A), concomitantly with modest enhancement of pAKT and pMTOR levels. However, pharmacological inhibition using AKTi or rapamycin failed to modulate LC3 expression in VPS4A‐silenced cells (Figure 4B), indicating that VPS4A silencing‐mediated autophagosome accumulation is an AKT/MTOR‐independent mechanism, likely by disrupting the final maturation or clearance of autophagosomes.

### 3.5 Alix Silencing Increases Lysosomal Degradation of Autophagosomes

To assess how Alix and VPS4A regulate the autophagic flux, we employed the mCherry‐GFP‐LC3 reporter system, in which GFP fluorescence is quenched in acidic lysosomes while mCherry remains stable. Alix silencing decreased the number of both mCherry^+^ and GFP^+^ puncta, indicative of impaired autophagosome biogenesis (Figure 4C). This is consistent with our earlier data showing LC3 reduction and AKT/MTOR activation. Surprisingly, despite the suppression of autophagosome formation, subsequent analyses revealed that Alix silencing enhanced lysosomal capacity. We observed an increased colocalization ratio of LC3 and LAMP1 (Figure 4D), and acridine orange (AO) and LysoTracker Red staining confirmed elevated lysosomal acidification (Figure 4E). To address the paradoxical observation of fewer autophagosomes being more efficiently targeted to lysosomes, we performed RAB7 co‐silencing and chloroquine (CQ) treatment, which blocks autophagosome‐lysosome fusion and lysosomal acidification. These interventions restored LC3 levels in Alix‐silenced cells, indicating that the autophagosomes are degraded through RAB7‐dependent fusion with hyperactive lysosomes (Figure 4F).

In contrast, VPS4A silencing caused an AKT/MTOR‐independent accumulation of LC3, which was also reversed by siRAB7 or CQ (Figure 4F), but without altering lysosomal acidification (Figure 4E). This indicates that VPS4A silencing induces abnormal autophagosome accumulation.

### 3.6 DN VPS4A Overexpression Impairs HBV Production via Lysosomes

**FIGURE 5:**
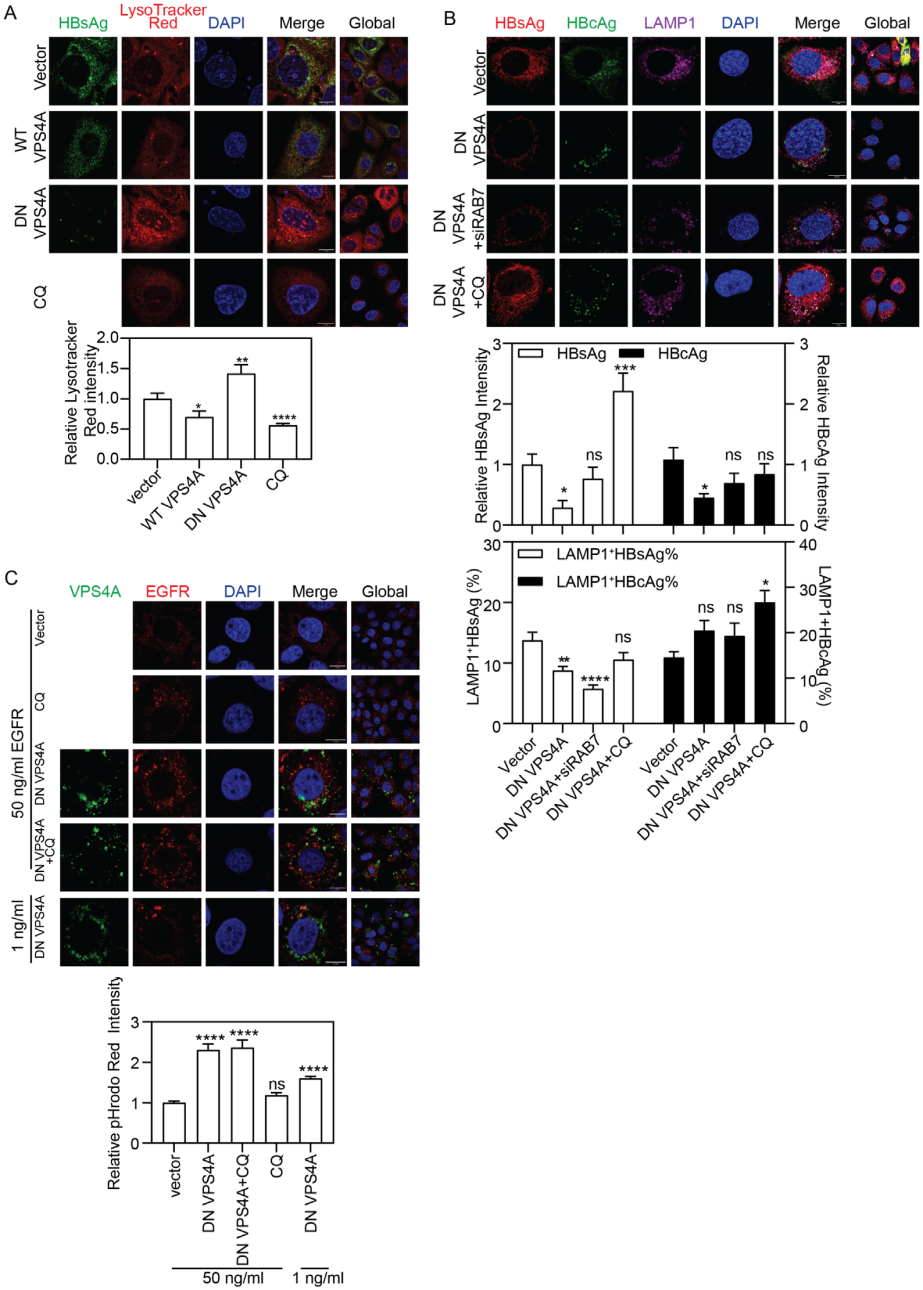
DN VPS4A overexpression reroutes protein trafficking. (A) LysoTracker Red intensity in Huh7 cells co‐transfected with pSM2 and control vector, WT, or DN VPS4A plasmids ± CQ. (B) HBsAg/HBcAg IF intensity and LAMP1 colocalization in pSM2‐transfected Huh7 cells under DN VPS4A overexpression ± CQ or siRab7. (C) pHrodo Red‐EGF fluorescence intensity (degraded EGFR) in Huh7 cells transfected with DN VPS4A ± CQ and pulsed with high (50 ng/mL) or low (1 ng/mL) EGF. Scale bar: 10 μm. *p < 0.05; **p < 0.01; ***p < 0.001; ****p < 0.0001; ns, not significant.

Having established that DN VPS4A severely suppresses HBV production, we sought to define the underlying mechanisms. We first confirmed that VPS4A specifically colocalizes to lysosomes, but not with markers of other organelles, indicating a specific functional role at the lysosome (Figure S5). We therefore hypothesized that DN VPS4A also affects lysosomal function. Indeed, LysoTracker staining revealed that DN VPS4A expression significantly enhanced lysosomal acidification compared to WT VPS4A or empty vector controls (Figure 5A). We next asked whether this enhanced lysosomal activity directly targets HBV antigens. Using a combination of RAB7 silencing and CQ treatment, we uncovered fundamentally distinct degradation pathways for HBsAg and HBcAg. For HBsAg, DN VPS4A markedly reduced both its total intracellular levels and its colocalization with lysosomes. This reduction was fully rescued by CQ, which restored HBsAg levels and its lysosomal association, but was unaffected by RAB7 silencing (Figure 5B). This defines a RAB7‐independent lysosomal degradation route for HBsAg that is strongly activated by DN VPS4A. As shown in Figure 3, DN VPS4A expression significantly increases the IF intensity of both LC3 and the late endosomal marker CD63, while markedly enhancing their colocalization with HBsAg. This suggests that when the canonical ESCRT‐mediated secretion pathway is paralyzed, HBV proteins are redirected into autophagosomes and late endosomes. Considering that DN VPS4A significantly increases the IF intensity of LC3 and CD63, along with their colocalization with HBsAg (Figure 3B,C), we propose that the paralysis of the canonical ESCRT‐mediated secretion pathway redirects HBV proteins into autophagosomes and late endosomes, leading to their subsequent degradation.

In contrast, HBcAg was eliminated via a different mechanism. DN VPS4A drastically reduced total HBcAg levels, and this reduction could not be rescued by either CQ or RAB7 silencing. However, CQ treatment under DN VPS4A expression caused a marked accumulation of HBcAg within lysosomes, an effect not seen with RAB7 silencing (Figure 5B). Thus, the clearance of HBcAg by DN VPS4A occurs through a pathway that is insensitive to lysosomal protease inhibition or RAB7‐mediated fusion, yet a subset of HBcAg is clearly capable of reaching the lysosomal compartment.

To evaluate whether the degradative function of DN VPS4A extends beyond HBV proteins, we examined its effect on the trafficking of epidermal growth factor receptor (EGFR), a canonical substrate for lysosomal degradation. Under low stimulation, receptors primarily localize to early endosomes for recycling, whereas high activation promotes lysosomal targeting [27]. Using pHrodo Red EGF conjugates that fluoresce in acidic compartments, we compared EGFR trafficking in DN VPS4A‐transfected Huh7 cells under 50 ng/mL (high) or 1 ng/mL (low) pHrodo Red EGF stimulation. Compared to control cells expressing an empty vector, transfection with DN VPS4A markedly enhanced red fluorescence under both high (50 ng/mL) and low (1 ng/mL) EGF stimulation, with the effect being more pronounced at the high concentration (Figure 5C), indicating DN VPS4A promotes EGFR trafficking to acidic compartments. Crucially, this intense fluorescence in DN VPS4A‐expressing cells persisted in the presence of CQ, whereas CQ treatment alone in control cells showed no significant signal enhancement. Since CQ inhibits lysosomal acidification but not late endosome acidification, these results demonstrate that DN VPS4A causes EGFR to accumulate specifically in acidic late endosomes, a compartment whose low pH is CQ‐insensitive. Thus, DN VPS4A disrupts the ESCRT‐dependent scission of vesicles into the lysosomal lumen, halting EGFR progression at the late endosomal stage.

### 3.7 L‐HBsAg Confers Resistance to DN VPS4A‐Mediated Degradation in the Lysosome

CQ combined with DN VPS4A selectively influenced HBsAg and HBcAg expression while leaving EGFR lysosomal degradation unaffected. Then we explored why certain HBV proteins are more susceptible to lysosomal degradation. We co‐transfected Huh7 cells with DN VPS4A and different HBsAg expression plasmids, including HBs‐2‐s (containing the HBV preS2 and S region with a low level expression) [28], HK188 (expressing HBV S protein at a high level) [29]. DN VPS4A significantly decreased HBsAg secretion, indicating DN VPS4A blocks HBsAg secretion independent of HBcAg (Figure S6).

**FIGURE 6:**
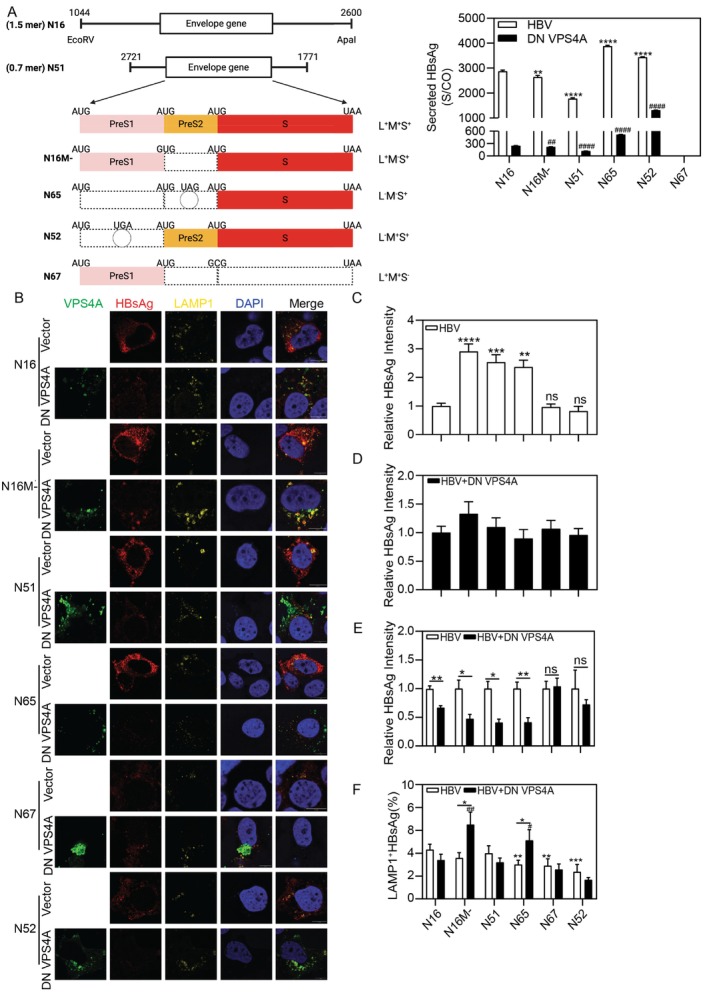
DN VPS4A directs S‐HBsAg degradation in lysosomes. Huh7 cells were co‐transfected with different HBV plasmids and control vector or DN VPS4A for 48 h. (A) The levels of HBsAg in the supernatants were measured using CMIA. (B) The IF intensity of HBsAg and its colocalization with LAMP1 were analyzed using ImageJ software. Scale bar: 10 μm. *p < 0.05; **p < 0.01; ***p < 0.001; ****p < 0.0001; ns, not significant.

We wonder whether different S‐, M‐, and L‐HBsAg compositions determine the route. Of the three envelope proteins produced by HBV, the L and S proteins are essential for virion assembly and secretion via the late endosomal pathway, whereas the M particle is dispensable [13]. Therefore, we transfected Huh 7 cells with the following HBV plasmids (Figure 6A), including 1.5× genome lengths (1.5mer, N16) expressing all three envelope proteins; 0.7× genome length (0.7mer, N51), which expresses three envelope proteins but lacks core protein; 1.5mer mutant expressing only L‐ and S‐HBsAg (N16M‐); constructs expressing only S‐HBsAg (N65), a combination of S and M HBsAg (N52), and only L‐HBsAg (N67) [30, 31] in the presence and absence of DN VPS4A plasmid. After 72 h, the supernatants were collected, and the HBsAg levels were determined. Compared to HBV plasmid transfection alone, DN VPS4A broadly reduced HBsAg secretion across all plasmid constructs, underscoring its strong inhibitory effects (Figure 6A). As an exception, L‐HBsAg is not secreted into the media and therefore cannot be detected using CMIA. We used IF staining and a confocal microscope to examine intracellular HBsAg distribution (Figure 6B). The baseline HBsAg levels varied across constructs (Figure 6C). The baseline intracellular HBsAg of N16M‐, N51, and N65 was higher than that of N16. However, there was no difference among N16, N67, and N52. Co‐transfection with DN VPS4A dampened the HBsAg staining intensity (Figure 6D). DN VPS4A co‐transfection significantly decreased intracellular HBsAg signal in cells expressing the L^+^M^−^S^+^ HBsAg combination (N16M‐), S‐HBsAg alone (N65). In stark contrast, the intensity of L‐HBsAg (N67) was completely unaffected by DN VPS4A, and the L^−^M^+^S^+^ HBsAg combination (N52) showed only a minor reduction (Figure 6E). This demonstrates that the susceptibility of intracellular HBsAg to DN VPS4A is strictly dependent on its protein composition. Crucially, this compositional specificity extended to lysosomal targeting. DN VPS4A significantly enhanced the colocalization of HBsAg with lysosomes exclusively in cells expressing L^+^M^−^S^+^ HBsAg combination (N16M‐) and S‐HBsAg alone (N65), but not in cells expressing L‐HBsAg alone or other combinations (Figure 6F). This result directly links the reduction in intracellular S‐HBsAg and L^+^M^−^S^+^ HBsAg to their diversion into the lysosomal pathway. Collectively, these data uncover a critical role for envelope protein composition in determining viral antigen fate under ESCRT impairment: S‐HBsAg is vulnerable to DN VPS4A‐induced lysosomal routing, while L‐HBsAg is less directed to lysosomes, and M‐HBsAg may partially shield S‐HBsAg from this degradation.

## 4 Discussion

Our study delineates distinct and complementary roles for the ESCRT‐associated proteins Alix and VPS4A in orchestrating the intracellular fate of HBV. We establish Alix as a critical regulator of early HBV sorting. Its depletion leads to the intracellular accumulation of viral components by impairing their entry into late endosomal and autophagic degradation pathways, while partially rerouting subviral particles toward secretion. In stark contrast, VPS4A primarily governs late‐stage endosomal trafficking and membrane scission. While its silencing has a relatively moderate impact and primarily promotes HBsAg degradation via the canonical autophagic pathway, the expression of DN‐VPS4A triggers a catastrophic collapse of the ESCRT machinery. This paralysis blocks canonical HBV egress and redirects viral proteins into late endosomes and autophagosomes for lysosomal degradation. These findings reveal that the ESCRT machinery acts as a central sorting switch, balancing HBV secretion against lysosomal eradication by precisely controlling the endosomal‐autophagic network.

A striking finding of our study is the cargo‐specific fate imposed by DN VPS4A on different viral and host proteins. This factor efficiently directed S‐HBsAg to lysosomes for degradation. It also triggered a rapid clearance of HBcAg, which might go through a pre‐lysosomal pathway. In contrast, L‐HBsAg remained entirely unaffected by DN VPS4A expression. This spectrum of outcomes demonstrates that VPS4A disruption does not cause a uniform blockade but rather is exquisitely sensitive to cargo identity. The resilience of L‐HBsAg is likely due to its unique transmembrane topology. These findings highlight how viral components have evolved distinct strategies to interface with the host transport machinery.

The mild phenotype observed upon siRNA‐mediated VPS4A knockdown, in contrast to the strong inhibition induced by DN VPS4A overexpression, can be explained by their distinct mechanisms of action. siRNA‐mediated knockdown reduces VPS4A protein levels but likely leaves a residual pool of functional protein, allowing limited ESCRT‐III disassembly and viral budding to proceed. In contrast, the DN VPS4A mutant acts dominantly by forming inactive complexes that sequester ESCRT‐III substrates, thereby stalling the entire machinery and profoundly disrupting MVB function. This mechanistic distinction accounts for the dramatic disruption in HBV trafficking, packaging, and replication seen only with the DN mutant.

While the broader requirement of ESCRT components in autophagosome maturation is recognized [32, 33], the specific role of Alix has remained less defined. Prior work identified an interaction between Alix and the ATG12‐ATG3 complex, implicating it in basal autophagosome formation [19]. Our study significantly extends this model. Besides the “binding partner” function, we also find that Alix silencing activates the AKT/MTOR signaling pathway, thereby suppressing autophagy initiation. To our knowledge, this is the first demonstration of Alix's roles in the AKT/MTOR signaling pathway. Furthermore, we found that Alix silencing concurrently improves lysosomal enzymatic activity and promotes the autophagosome‐lysosome fusion. The potential involvement of Alix in autophagosome closure remains unresolved. While other ESCRT components like VPS37A [34] and CHMP2A [17] have been implicated in phagophore closure, our observation that Alix silencing increases LC3‐LAMP1 colocalization suggests it is not essential for this step in our system. This is further supported by recent evidence indicating that autophagosome closure may not be a strict prerequisite for lysosomal fusion [35]. Consequently, the functional contribution of Alix to autophagosome closure regulation remains ambiguous and needs further investigation.

Physiologically, the nutrient‐dependent role of Alix in autophagy may be subverted by the virus. Chronic HBV infection has been shown to alter the host's metabolic landscape [36]. In such a perturbed metabolic milieu, the virus could potentially exploit Alix‐mediated suppression of basal autophagy to limit the degradation of viral components and facilitate its persistence. Conversely, the potent degradation‐promoting effects of DN VPS4A demonstrate that forcibly shifting the ESCRT‐controlled balance toward lysosomal degradation can counteract viral evasion mechanisms. While VPS4A is known to maintain lysosomal morphology [37] and facilitate membrane repair [38], how its dominant‐negative mutant upregulates lysosomal enzymatic activity remains an important question for future study.

In summary, this study highlights the intricate roles of Alix and VPS4A in HBV replication. Our results demonstrate that they critically influence HBV trafficking between endosomes and autophagosomes, with specific mechanisms regulating autophagosome formation and lysosomal degradation of viral components. By advancing our understanding of these complex interactions, this work sheds new light on the host‐cell pathways that control HBV replication and persistence.

## Author Contributions

Jia Li: writing – original draft, writing – review and editing, visualization, investigation, data curation, methodology, formal analysis. Thekla Kemper: methodology. Shuping Tong: review and editing, supervision, resources. Xueyue Wang and Yong Lin: review and editing. Mengji Lu: writing – review and editing, supervision, resources, project administration, funding acquisition, formal analysis.

## Funding

This work was supported by grants of Deutsche Forschungsgemeinschaft to M. L. (Grants Lu 669/12‐1 and Lu 669/13‐1).

## Conflicts of Interest

The authors declare no conflicts of interest.

## Supporting information


**Table S1:** List of plasmids.
**Table S2:** List of antibodies.

**Figure S1:** HBV upregulates the expression levels of Alix and VPS4A.
**Figure S2:** The silencing efficiency of siAlix and siVPS4A.
**Figure S3:** Alix and VPS4A silencing yield similar results in pSM2‐transfected Huh7 cells as compared with those in HepG2.2.15 cells.
**Figure S4:** The effects of Alix and VPS4A silencing on the HBV trafficking along endosomal and autophagic pathways.
**Figure S5:** VPS4A primarily colocalizes with LAMP1.
**Figure S6:** DN VPS4A blocks HBsAg secretion is independent of HBcAg.

## Data Availability

Data sharing not applicable to this article as no datasets were generated or analyzed during the current study.
