# Supplementary material for: The Roles of Alix and VPS4A in Autophagy and Endosomal Pathways and Their Relation to HBV Replication
Source: FASEB J. 2026 Apr 8;40(7):e71771. doi: 10.1096/fj.202504742R (PMC13060583; doi:10.1096/fj.202504742R)
Supplement: Supplementary file 1 — Table S1: List of plasmids. Table S2: List of antibodies. [file FSB2-40-e71771-s001.docx]

**Table S1.** List of plasmids.

| Name | Source |
| --- | --- |
| pSM2 | Stored in our laboratory |
| mCherry-GFP-LC3 | Stored in our laboratory |
| pUC19 vector | Stored in our laboratory |
| N16 (1.5x L+M+S+) | Stored in our laboratory |
| N16M- (1.5x L+M-S+) | Stored in our laboratory |
| N51 (0.7x L+M+S+) | Stored in our laboratory |
| N65 (0.7x L-M-S+) | Stored in our laboratory |
| N67 (0.7x L+M+S-) | Stored in our laboratory |
| N52 (0.7x L-M+S+) | Stored in our laboratory |
| HK188 | Stored in our laboratory |
| HBs-2-s | Stored in our laboratory |
| S-HBsAg  S-HBsAg N146- | Stored in our laboratory  Stored in our laboratory |
| pCINeoFlag Alix | Addgene, 89859; deposited by Wesley Sundquist |
| pCINeoFlag Alix; I212D | Addgene, 89861; deposited by Wesley Sundquist |
| pDONR 223 VPS4A | Addgene, 169319; deposited by Francisca Vazquez |
| pEGFP-VSP4-E228Q | Addgene, 80351; deposited by Wesley Sundquist |
| E228Q Vps4A-HA | Addgene, 200087; deposited by Jason MacGurn |
|  |  |

**Table S2.** List of antibodies.

| Product Name | Company |
| --- | --- |
| Anti-Alix Rabbit antibody | Cell Signaling Technology, 92880S |
| Anti-VPS4A Mouse antibody | Santa Cruz Biotechnology, sc-393428 |
| Anti-PDI Rabbit antibody | Cell Signaling Technology, 3501S |
| Anti-GM130 Rabbit antibody | Cell Signaling Technology, 12480S |
| Anti-RAB5A Rabbit antibody | Cell Signaling Technology, 46449S |
| Anti-CD63 Mouse antibody | Santa Cruz Biotechnology, sc-5275 |
| Anti-LAMP1 Mouse antibody | Cell Signaling Technology, 15665S |
| Anti-LC3B Rabbit antibody | Cell Signaling Technology, 3868S |
| Anti-p62 Rabbit antibody | Cell Signaling Technology, 5114S |
| Anti-ACTB Mouse antibody | Sigma-Aldrich, A5441 |
| Anti-HBsAg Mouse antibody | ZSGB-BIO, ZM-0122 |
| Anti-HBcAg Rabbit antibody | ZSGB-BIO, ZA-0121 |
| Anti-AKT Rabbit antibody | Cell Signaling Technology, 9272S |
| Anti-Phospho-AKT Rabbit antibody | Cell Signaling Technology, 9271S |
| Anti-MTOR Rabbit antibody | Cell Signaling Technology, 2972S |
| Anti-Phospho-MTOR Rabbit antibody | Cell Signaling Technology, 2971S |
| Anti-RPS6KB Rabbit antibody | Cell Signaling Technology, 9202S |
| Anti-Phospho-RPS6KB Rabbit antibody | Cell Signaling Technology, 9209S |
