# Supplementary material for: The Roles of Alix and VPS4A in Autophagy and Endosomal Pathways and Their Relation to HBV Replication
Source: FASEB J. 2026 Apr 8;40(7):e71771. doi: 10.1096/fj.202504742R (PMC13060583; doi:10.1096/fj.202504742R)
Supplement: Supplementary file 2 — Figure S1: HBV upregulates the expression levels of Alix and VPS4A. Figure S2: The silencing efficiency of siAlix and siVPS4A. Figure S3: Alix and VPS4A silencing yield similar results in pSM2‐transfected Huh7 cells as compared with those in HepG2.2.15 cells. Figure S4: The effects of Alix and VPS4A silencing on the HBV trafficking along endosomal and autophagic pathways. Figure S5: VPS4A primarily colocalizes with LAMP1. Figure S6: DN VPS4A blocks HBsAg secretion is independent of HBcAg. [file FSB2-40-e71771-s002.docx]

# Supplemental Figures

Fig. S1. HBV upregulates the expression levels of Alix and VPS4A.


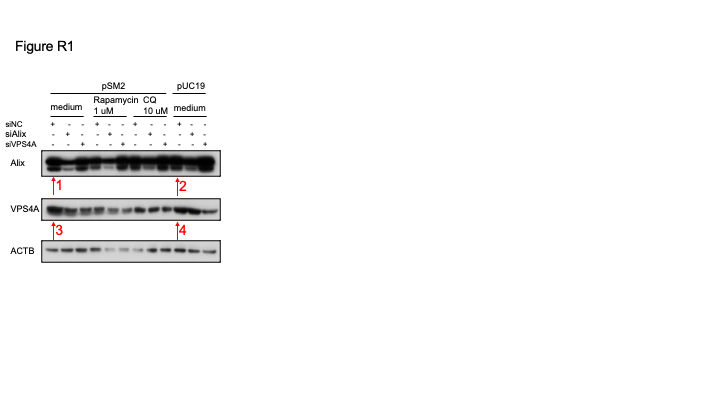


Huh7 cells were transfected with either an HBV expression plasmid (pSM2) or an empty vector control (pUC19) for 48 hours. Representative Western blot lanes showing the protein levels of Alix and VPS4A, with ACTB as the internal control. HBV expression led to a 1.15-fold (15%) increase in Alix levels (OD: Lane1_1.89 vs. Lane2_1.64) and a 1.40-fold (40%) increase in VPS4A levels (OD: Lane3_1.88 vs. Lane4_1.34) compared to the mock control.

Fig. S2. The silencing efficiency of siAlix and siVPS4A.


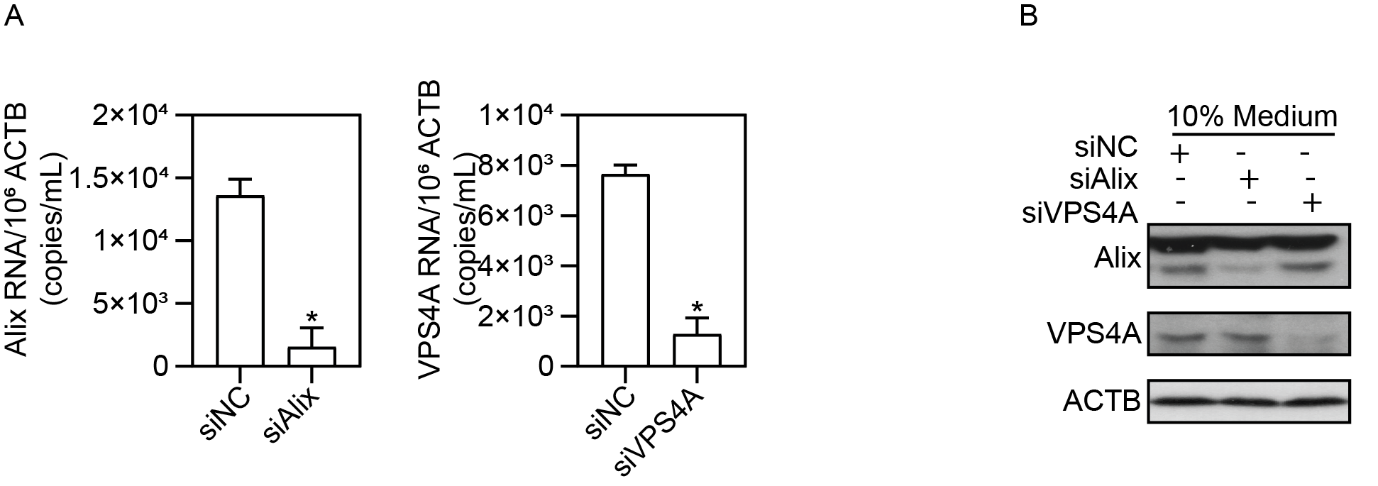


Huh7 cells were co-transfected with pSM2 and siAlix, siVPS4A, or siNC, and harvested after 72 h. (**A**) The RNA levels of Alix and VPS4A were measured by real-time PCR, and (**B**) protein levels were measured by western blot.

Fig. S3. Alix and VPS4A silencing yield similar results in pSM2-transfected Huh7 cells as compared with those in HepG2.2.15 cells.


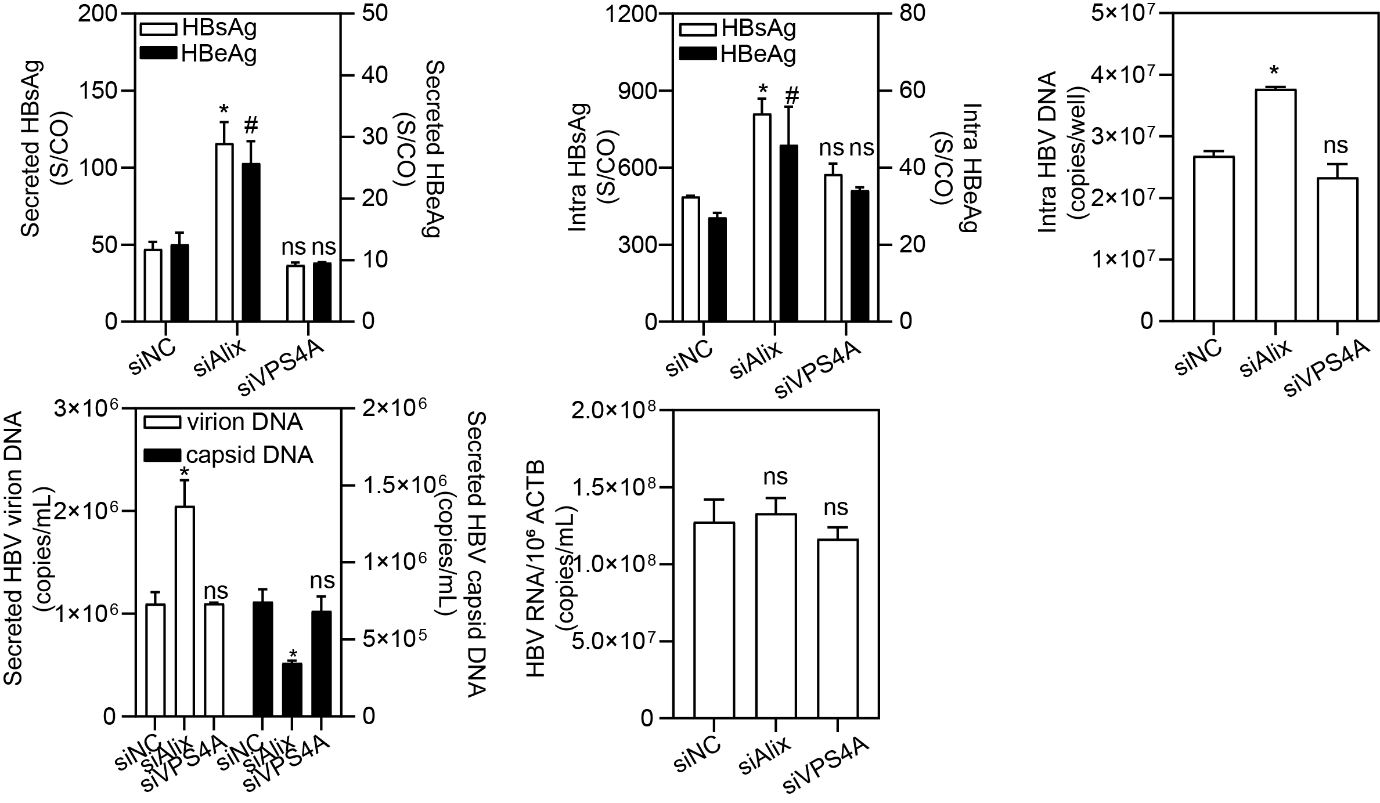


Huh7 cells were co-transfected with pSM2 and siAlix, siVPS4A, or siNC, respectively, and harvested after 72 h. The levels of HBsAg and HBeAg in the supernatants and cell lysates were measured using CMIA. HBV DNA levels in cell lysates were quantified by qPCR. Virions and capsids in supernatant obtained from immunoprecipitates using HBsAg and HBcAg antibodies were quantified by qPCR. Intracellular HBV total RNA levels were determined by RT-PCR.

Fig. S4. The effects of Alix and VPS4A silencing on the HBV trafficking along endosomal and autophagic pathways.


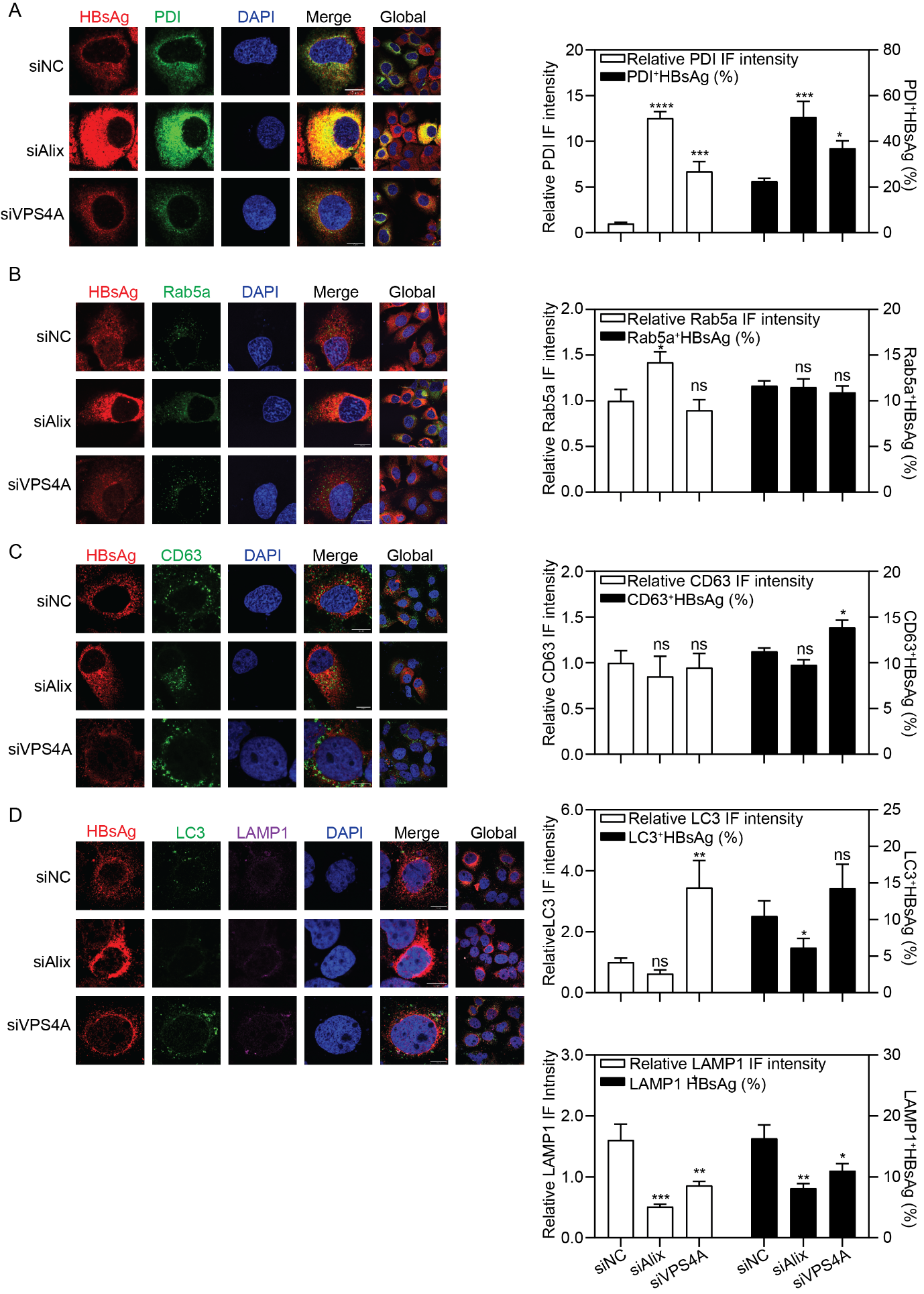


Huh7 cells co-transfected with pSM2 and siAlix, siVPS4A, or siNC, respectively, and harvested after 72 h. The colocalization of (**A**) PDI, (**B**) RAB5A, (**C**) CD63 with HBsAg, (**D**) LC3 with HBsAg or LAMP1 were detected. The fluorescence intensity of target proteins and the colocalization between target proteins were analyzed using ImageJ software. Scale bar: 10 μm. ^*^p < 0.05; ^**^p < 0.01; ^***^p < 0.01; ^****^p < 0.0001; and ns, not significant.

The effects of siAlix and siVPS4A on HBV trafficking were further assessed in Huh7 cells. Huh7 cells were transiently co-transfected with pSM2 and siAlix or siVPS4A for 48 h, and the expression levels of PDI, RAB5A, CD63, LC3, and LAMP1, as well as their colocalization with HBsAg, were analyzed. Consistent with observations in HepG2.2.15 cells, Alix silencing increased PDI expression and its association with HBsAg (Fig. S3A). However, in contrast to HepG2.2.15 cells, where RAB5A^+^HBsAg% increased from 5% to 15%, Alix silencing in Huh7 cells did not affect RAB5A colocalization with HBsAg (Fig. S3B). The baseline RAB5A^+^HBsAg% in Huh7 cells was approximately 15%, suggesting that it may have already reached its maximum and could not increase further. Similarly, Alix silencing did not significantly affect CD63 expression or its colocalization with HBsAg (Fig. S3C). While siAlix reduced CD63^+^HBsAg% from 15% to 10% in HepG2.2.15 cells, it caused a minor decrease from 12% to 9% in Huh7 cells, consistent with a baseline near the minimum threshold. LC3 expression was slightly decreased, though its colocalization with HBsAg was significantly reduced (Fig. S3D). This reduction was less pronounced in Huh7 cells due to the already low baseline levels of LC3. Consistent with the results in HepG2.2.15 cells, Alix silencing decreased LAMP1 expression and its colocalization with HBsAg (Fig. S3D).

Unlike in HepG2.2.15 cells, VPS4A silencing significantly increased PDI expression and its association with HBsAg (Fig. S3A) but significantly reduced LAMP1 expression and its colocalization with HBsAg in Huh7 cells (Fig. S3D). Aligning with findings in HepG2.2.15 cells, VPS4A silencing did not alter RAB5A expression or its colocalization with HBsAg (Fig. S3B). CD63 expression was unaltered, though its colocalization with HBsAg increased (Fig. S3C). VPS4A silencing also increased LC3 colocalization with HBsAg (Fig. S3D).

Despite some discrepancies between the stable HBV replication system (HepG2.2.15) and the transient de novo replication model (Huh7), the results consistently demonstrate that Alix silencing increases HBsAg accumulation in the ER while reducing its trafficking to autophagosomes, late endosomes, and lysosomes. In contrast, VPS4A silencing had minimal effects on HBsAg trafficking but delayed HBsAg secretion.

Fig. S5. VPS4A primarily colocalizes with LAMP1.


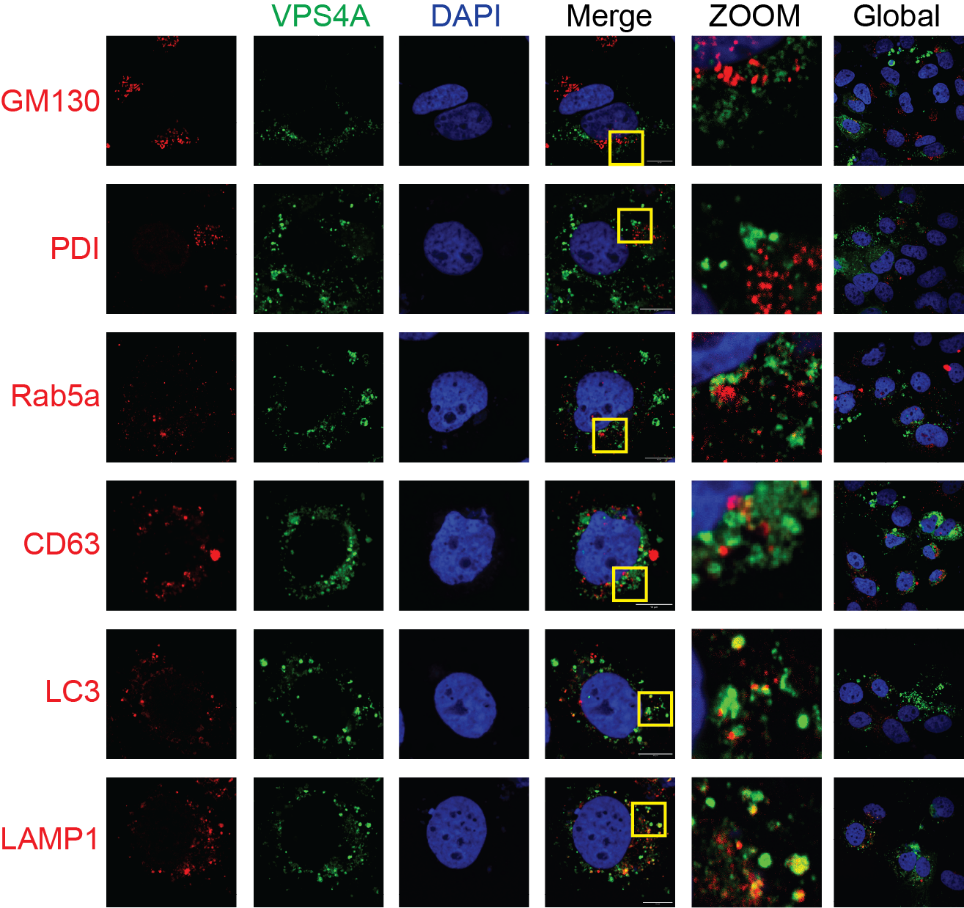


Huh7 cells were transfected with DN VPS4A for 48 hours. The distribution of VPS4A and GM130, PDI, Rab5a, CD63, LC3, and LAMP1 were analyzed using ImageJ software.

Figure S6. DN VPS4A blocks HBsAg secretion is independent of HBcAg.


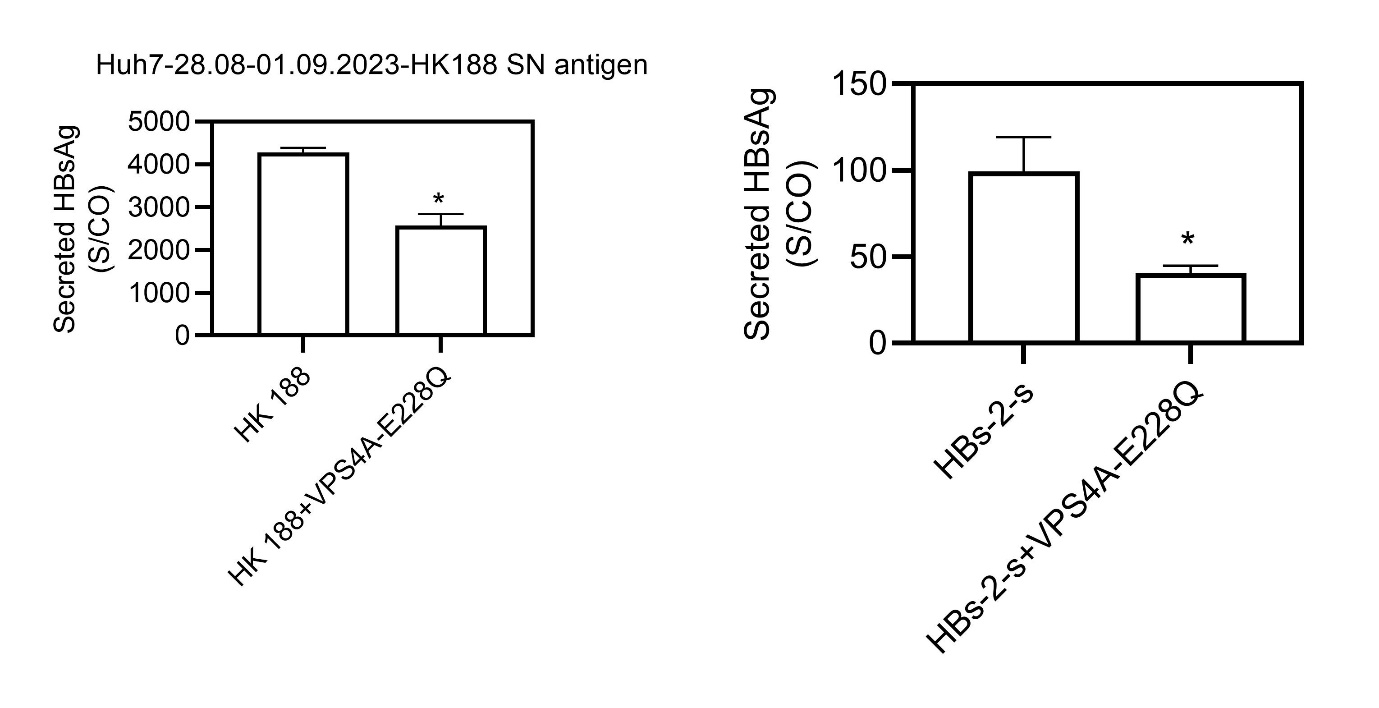

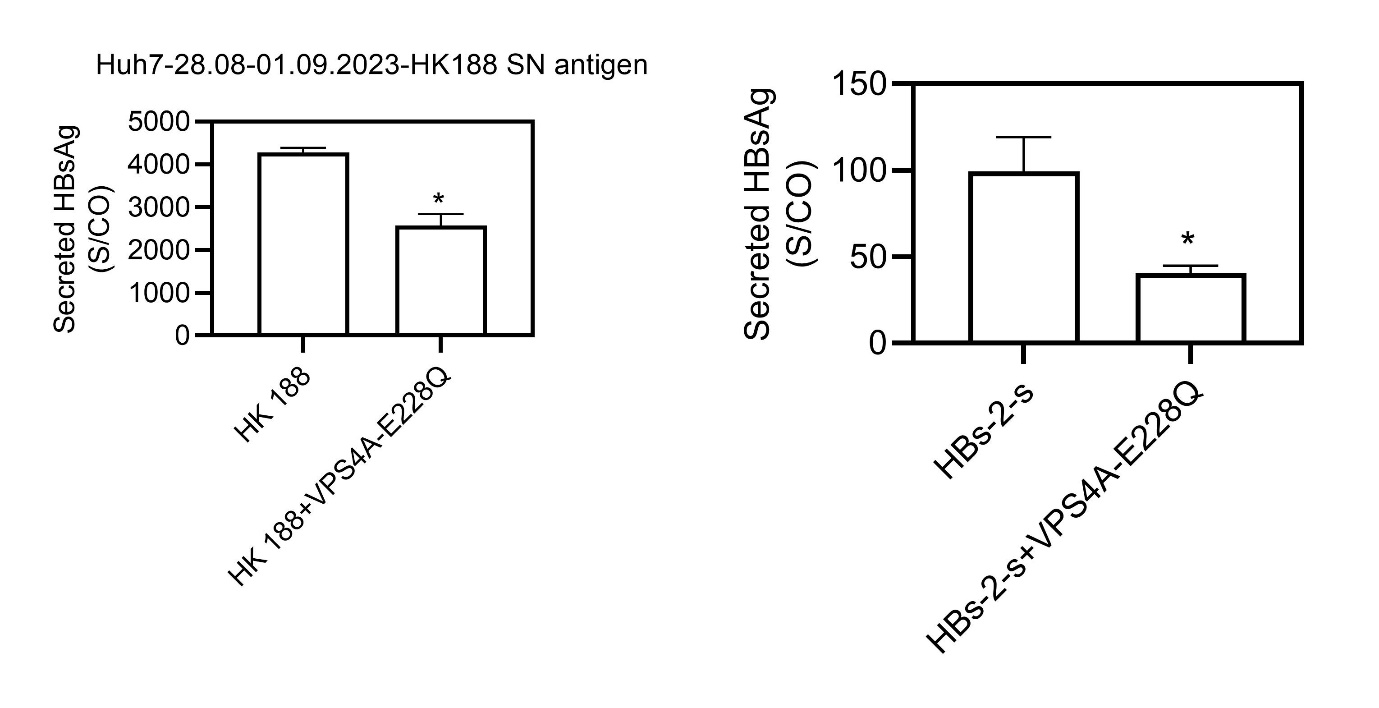
Huh7 cells co-transfected with DN VPS4A and HK188 and HBs-2-s, respectively, for 72 h. The levels of HBsAg in the supernatants were measured using CMIA.
